# Not so different after all? Comparing patients attending general practice-based locally enhanced services for sexual health with patients attending genitourinary medicine

**DOI:** 10.1177/0956462412472301

**Published:** 2013-02

**Authors:** C H Mercer, C R H Aicken, J A Cassell, V Hartnell, L Davies, J Ryan, F Keane

**Affiliations:** * UCL Centre for Sexual Health & HIV Research, 3rd floor Mortimer Market Centre off Capper Street, London WC1E 6JB; † Brighton & Sussex Medical School, Brighton; ‡ Falmouth Health Centre Practice; § Helston Medical Centre; ** Alverton Practice; †† Royal Cornwall Hospitals, Cornwall, UK

**Keywords:** genitourinary medicine clinic, primary care, general practice, locally enhanced service, service delivery, sexual behaviour, sexually transmitted infections, access, survey, UK

## Abstract

We did a cross-sectional survey of patients attending genitourinary (GU) medicine clinics (*n* = 933) and general practice-based Locally Enhanced Services for Sexual Health (GP-LESSH, *n* = 111) in Cornwall, England, in 2009/2010, to compare patients’ characteristics and experiences. Patients completed a pen-and-paper questionnaire that was then linked to an extract of their clinical data. GP-LESSH patients took longer both to seek and to receive care: medians of nine and seven days, respectively, versus GU medicine patients: medians of seven and one day, respectively. GP-LESSH patients were less likely than GU medicine patients to report symptoms (19.6% versus 30.6%) and sexual risk behaviours (33.3% versus 44.7% reported new partners) since recognizing needing to seek care; 5.0% versus 10.2% were men who have sex with men). However, they were equally likely to have sexually transmitted infections (STIs) diagnosed (23.3% versus 24.8%). As GP-LESSH may operate infrequently, local services must work collaboratively to ensure that those seeking care for suspected STIs receive it promptly. Failing to do so facilitates avoidable STI transmission.

## INTRODUCTION

There have been substantial changes in the delivery of sexual health care in England over the past decade reflecting national guidance^1–3^ and targets^4^ focused on improving access to sexual health care. Reflecting this strategic vision, services for sexually transmitted infections (STIs) are increasingly available outside the specialist genitourinary (GU) medicine clinic setting in community settings, including through the National Chlamydia Screening Programme (NCSP).^5^ Commissioning models introduced in the early 2000s encouraged the development of more specialized sexual health provision within primary care, including ‘Locally Enhanced Services’ for sexual health (LESSH). Although transferable staff competencies for STI management have been developed,^6–9^ these are not universally provided in LESSH^10^ and there are variations in the structure and scope of LESSH and their effectiveness in STI control.^11^ In some areas, all LESSH regardless of setting, provide a specified locally standardized service, but in others, LESHH perform only minimal sexual health activities,^12^ such as condom distribution.

It is unclear which models of LESSH are most cost-effective for disease control, and guidance for service planners does not address how best to commission a range of GU medicine and LESSH to maximize public health gain.^13^ There is thus an urgent need for service planners and providers to understand the risk profiles and care pathways of people who seek care for suspected STIs in their locality so as to optimize the capability of services to meet individual needs, as well as public health needs.

While GU medicine clinics routinely report surveillance data on attendances, diagnoses and access,^14,15^ little is known about people seeking care from community-based services.^16^ Here we report the results of a survey tool designed to supplement routinely collected clinical data, administered in GU medicine clinics and nearby general practice-based LESSH (GP-LESSH), to compare sociodemographic and behavioural characteristics and care pathways of patients attending these services in one area of England: Cornwall.

## METHODS

### Setting

We undertook a comparative cross-sectional survey of attendees at GU medicine clinics and GP-LESSH in Cornwall, a largely rural area in southwest England. Data collection ran from 2 November 2009 to 24 December 2009 in GU medicine and from 10 February 2010 to 29 April 2010 in GP-LESSH. The rationale and design of this survey, together with the study materials, have previously been published.^17^

The main GU medicine clinic opened every working day, running both appointment and walk-in clinics, and had four satellite GU medicine clinics. These offered a mixture of walk-in and appointment clinics: three operated on at least a weekly basis and one fortnightly. Access to all clinics is coordinated centrally and a walk-in facility is available at one location every working day.

LESSH are provided in three types of service in Cornwall: the majority in general practices, one Brook Advisory Centre (young people's clinic), and a community hospital. We surveyed all three GP-LESSH services, each aimed to provide appointments to at least 10 new patients per week, as separate timed clinics rather than dispersed appointments throughout the week.

Reception staff at both settings were asked to distribute pen-and-paper questionnaires to all patients. These asked about reason(s) for attendance, whether the patient had symptoms, when they first sought care and where, experience of other services, and sexual behaviour questions.

Questionnaires were anonymous apart from the patient's clinic number. Respondents were asked to consent to linkage of their questionnaire to an extract of clinical data including tests done, STI diagnosis/es made, and additionally (for GP-LESSH patients), treatment(s) and/or referral. The latter were coded for appropriateness of care (Supplementary Table S3; please see http://std.rsmjournals.com/lookup/suppl/doi:10.1177/0956462472301/-/DC1).

Using aggregate-level data available for both GU medicine^14^ and GP-LESSHs in Cornwall (part of Cornwall's LESSH requirements), we compared our patient sample to the patient population with respect to gender, age, ethnicity and whether or not STI(s) were diagnosed.

### Statistical analysis

We calculated percentages and percentiles to describe the sample of patients, stratified by the type of service attended (GU medicine versus GP-LESSH) and gender, and used the chi-squared statistic to identify differences between these groups. Analyses were undertaken using the survey commands in Stata 10.0^18^ to take account of clustering of patients by clinic/practice. Statistical significance was considered as *P* < 0.05 for all analyses.

Sample size calculations were not undertaken reflecting the principal aim of the parent study to develop and demonstrate an audit tool capable of gathering epidemiological data rapidly to inform service planning.^17^

### Ethical approval

The research protocol was approved by the London Research Ethics Committee (number: 09/H0718/1).

## RESULTS

### Response rates

Altogether, 933 GU medicine patients completed the questionnaire (one-third, 319, doing so at a satellite GU medicine clinic); 111 patients completed the questionnaire in GP-LESSH. Response rates were similar: 57.4% in GU medicine overall (range: 53.4–76.1%) versus 51.1% in GP-LESSH overall (range: 37.2–62.9%), and similar proportions of respondents consented to linkage (83.1% versus 81.3%, respectively). There were no differences between patients who consented to linkage and those who did not (data not shown). GU medicine respondents were slightly younger than the whole GU medicine patient population with 50.6% aged under 25 (versus 44.5%, *P* = 0.001), while similar comparisons for GP-LESSH found no difference.

### Comparison of GU medicine versus GP-LESSH patients

#### Sociodemographics

Similar proportions of patients in the GU medicine and GP-LESSH samples were female (57.5% versus 61.3%, respectively), and the median age of respondents was also similar (24 versus 23 years, respectively, Supplementary Table S1; please see http://std.rsmjournals.com/lookup/suppl/doi:10.1177/0956462472301/-/DC1). However, male GU medicine respondents were older on average than male GP-LESSH respondents (medians: 27 versus 24 years, respectively), the latter being similar in age to their female counterparts. Reflecting the local demography, nearly all respondents were of white ethnicity.

#### Sexual behaviours

While nearly all (94.1%) patients surveyed in both settings reported exclusively opposite-sex partner(s) in the past year (Supplementary Table S1), 10.2% of male GU medicine respondents reported same-sex partner(s), contrasting with 5.0% of men in the GP-LESSH sample. The median number of partners in the past year was two among both GU medicine and GP-LESSH patients; however, one-tenth of men attending GU medicine reported 10 or more partners in this time-frame versus none of the men attending GP-LESSH and less than 2% of all women studied. This pattern persisted when the denominator was limited to those reporting only opposite-sex partner(s) in the past year (data not shown). GU medicine respondents more often reported new partner(s) in the past year (80.8% versus 73.3%, respectively), as well as *multiple* new partners in this time frame, at least among men (55.8% versus 45.2%). While the median number of partners in the last three months was one for all gender/service groups, men in the GU medicine sample were more likely to report at least two partners in this time frame (36.4% versus 19.1%, respectively).

#### Health-related factors

Nearly 90% of all respondents attended for a new episode of care (Supplementary Table S1). Two-thirds (67.4%) of men in the GP-LESSH sample had never attended the service before, a higher proportion than in the GU medicine sample (46.0%).

Nine-tenths of patients in both settings reported registration with a GP, although among men attending GP-LESSH this was lower, with a quarter responding either ‘no’ or ‘I'm not sure’ to this question. Females in both settings were more likely than males to report previous chlamydia testing (82.5% versus 65.0% of all women and men, respectively). However, there was no difference by gender or setting in the proportion reporting previous STI diagnosis/es (approximately 1 in 3 patients surveyed).

#### Reasons for seeking care

The two most commonly cited reasons for seeking care were the same in the two settings: having symptoms (38.4%) or not having symptoms but wanting a check-up (37.5%), accounting for three-quarters of respondents (Supplementary Table S2; please see http://std.rsmjournals.com/lookup/suppl/doi:10.1177/0956462472301/-/DC1). However, more GP-LESSH patients than GU medicine patients reported seeking care because they wanted an asymptomatic check-up (47.6% versus 36.3%), while a slightly larger proportion of GU medicine patients reported wanting an HIV test as their reason for attendance (7.1% versus 4.9%).

#### Accessing care

**Figure 1:**
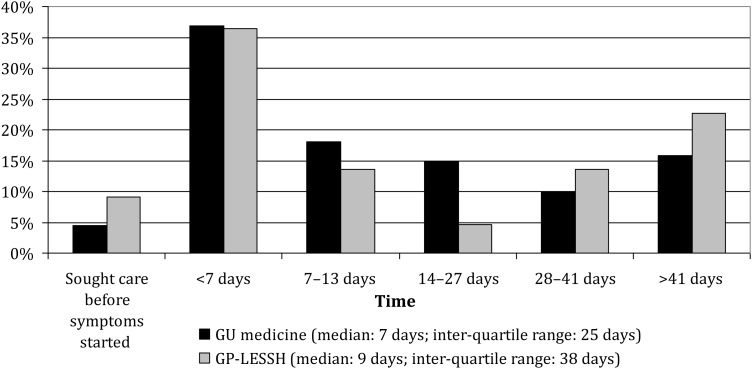
Distribution of time between recognizing symptoms (denominator is limited to those reporting symptoms: 30.6% of GU medicine patients [*n* = 266]; 19.6% of GP-LESSH patients [*n* = 22]) and first seeking care by setting (GU medicine clinic versus GP-LESSH) (No statistically significant gender difference in either settings). GU = genitourinary; GP-LESSH = general practice-based Locally Enhanced Services for Sexual Health

**Figure 2:**
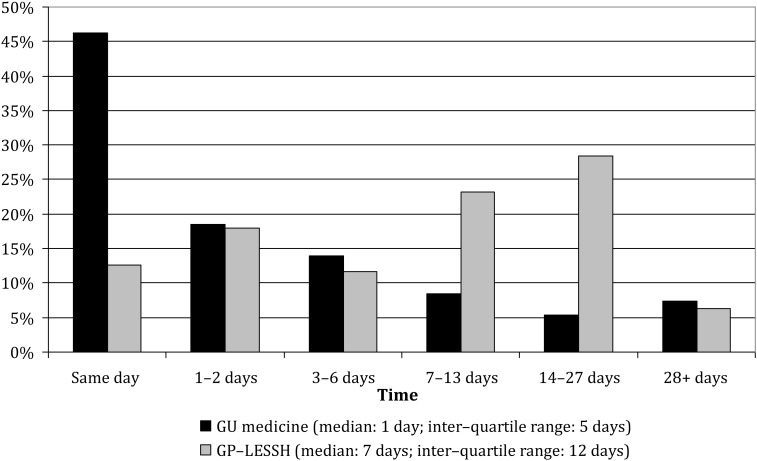
Distribution of time since first seeking care from any health-care service and being seen in the study clinic/practice by setting (GU medicine clinic versus GP-LESSH) (No statistically significant gender difference in either setting). Denominator is all patients: 933 GU medicine patients and 111 GP-LESSH patients. GU = genitourinary; GP-LESSH = general practice-based Locally Enhanced Services for Sexual Health

GU medicine patients more often reported symptoms at the time of their consultation (30.6% versus 19.6%, respectively), and among symptomatic patients, the median time between recognizing a need to seek care and first trying to do so was seven days among GU medicine patients and nine days among GP-LESSH patients (Figure 1). Approximately a quarter of GU medicine patients (24.4%) reported first using, or trying to use, another health-care service, which in two-thirds of cases was general practice. This finding was unchanged when patients attending satellite GU medicine clinics were excluded (data not shown). A smaller proportion of GP-LESSH patients (9.5%) reported trying to use another health-care service first, usually another GP. Once patients sought care, GU medicine respondents received care more promptly: 46.3% were seen on the day that they first sought care compared with 12.6% of GP-LESSH patients (Figure 2).

#### Sex since recognizing a need to seek care

Despite their longer care pathway, GP-LESSH respondents were equally likely as GU medicine respondents to report sex since recognizing a need to seek care (40.7% of all patients, Table 1). However, fewer male GU medicine respondents reported sex during this time than male GP-LESSH respondents (33.7% versus 45.0%, respectively); while among women the converse was evident (46.1% versus 33.9%, respectively). Over 80% of all respondents reporting sex while seeking care reported just one partner during this time, although more GU medicine patients reported multiple partners: 20.0% versus 7.5%, with this difference more marked for men (19.5% versus 5.6%) than women (20.3% versus 9.1%, respectively).

**Table 1:** A comparison of patients’ sexual risk behaviour since recognizing a need to seek care by setting (GU medicine clinic versus GP-LESSH) and gender

Gender	Males and females			Males			Females		
Setting:	GU medicine	GP-LESSH	*P* value*	GU medicine	GP-LESSH	*P* value*	GU medicine	GP-LESSH	*P* value*
*Denominator* ^†^	*933*	*111*		*397*	*43*		*536*	*68*	
Had sex since recognized a need to seek care	41.0%	38.1%	0.617	33.7%	45.0%	0.061	46.1%	33.9%	0.854
*Denominator* ^‡,§^ *:*	*363*	*40*		*124*	***18***		*239*	***22***	
2+ partners since recognized a need to seek care	20.0%	7.5%	0.067	19.5%	**5.6%**	**0.022**	20.3%	**9.1%**	**0.154**
Any *new* partners since recognized a need to seek care	44.7%	33.3%	<0.001	42.7%	**27.8%**	**0.091**	45.7%	**38.1%**	**0.307**
Median number of sex acts since recognized a need to seek care (lower, upper quartiles)	4 (2, 10)	4 (1, 10)	0.991	4 (2, 10)	**7 (3, 12)**	**0.306**	3 (2, 8)	**2 (1, 10)**	**0.470**
Condom use since recognized a need to seek care			0.417			**0.562**			**0.369**
Every time	19.8%	27.5%		28.5%	**33.3%**		15.0%	**22.7%**	
Most times	8.0%	2.5%		9.8%	**0.0%**		7.1%	**4.6%**	
Half of the time	8.0%	0.0%		7.3%	**0.0%**		8.4%	**0.0%**	
Sometimes	10.6%	25.0%		10.6%	**16.7%**		10.6%	**31.8%**	
Not at all	53.6%	45.0%		43.9%	**50.0%**		58.9%	**40.9%**	

GU = genitourinary; GP-LESSH = general practice-based Locally Enhanced Services for Sexual Health

*P value for difference between settings

^†^Among all patients

^‡^Among patients reporting sex since recognizing a need to seek care

^§^Estimates are shown in bold to denote that caution is needed with their interpretation due to small denominators

GU medicine respondents were also more likely than GP-LESSH respondents to report *new* partner(s) since recognizing a need to seek care: 44.7% versus 33.3%, with again, a more marked difference among men (42.7% versus 27.8%) than women (45.7% versus 38.1%). However, both GU medicine patients and GP-LESSH patients reported a median of four sex acts since recognizing a need to seek care (interquartile range: 2–10). Over three-quarters of these reported *un*protected sex at least once, a proportion that was larger among GU medicine patients than GP-LESSH patients (80.2% versus 72.5%). GU medicine patients were more likely than GP-LESSH patients to report not using condoms at all during this time (53.6% versus 45.0%, respectively).

#### Testing for STIs

**Figure 3:**
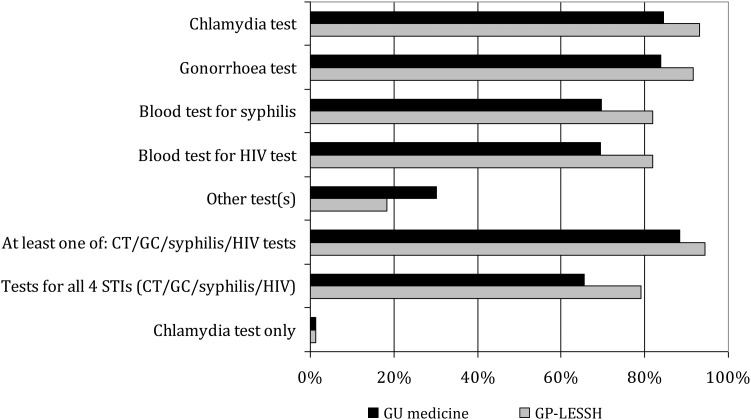
STI tests received by patients attending for a new episode of care (denominator excludes patients reporting attending for a follow-up appointment, leaving: 585 GU medicine patients and 72 GP-LESSH patients, thus numbers of GP-LESSH patients too small to permit analyses by gender) reporting reason(s) suggestive of an STI (among patients reporting 1+ of the following reason(s) for attendance: ‘I have (or had) symptoms’, ‘My partner has (or had) symptoms’, ‘I did not have symptoms but wanted a check-up’, ‘My partner has been diagnosed with an infection and needed to come to the clinic/surgery’, ‘Someone from the clinic/surgery called me in’, ‘My GP or practice nurse told me to come here’) by setting (GU medicine clinic versus GP-LESSH). GU = genitourinary; GP-LESSH = general practice-based Locally Enhanced Services for Sexual Health; STI = sexually transmitted infection; CT = *Chlamydia trachomatis*; GC = gonorrhoea

In both settings, almost all (94.1%) patients attending for a new episode of care who were attending for a suspected STI (see footnotes to Figure 3) had a genital examination. Similarly, almost all were tested for at least one of chlamydia, gonorrhoea, syphilis and/or HIV (Figure 3). This proportion was slightly larger among GP-LESSH patients (94.4% versus 88.4%) but GP-LESSH patients were more likely than GU medicine patients to be new patients rather than attending for a new episode of care or a follow-up appointment. Very few (1.2%) tested just for chlamydia. Indeed, 79.2% of GP-LESSH respondents and 65.5% of GU medicine respondents were recorded as testing for chlamydia, gonorrhoea, syphilis *and* HIV, although gender differences were evident in both settings: 96.3% versus 68.9% for men and women attending GP-LESSH, respectively; 74.4%, versus 59.1% for men and women attending GU medicine, respectively.

#### STI diagnosis/es

**Figure 4:**
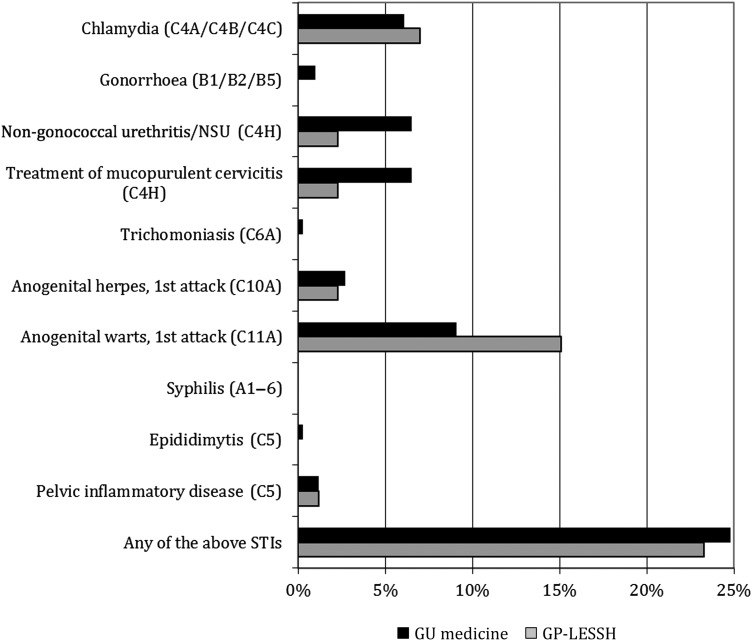
Percent of patients diagnosed with STIs during their episode of care by setting (GU medicine clinic versus GP-LESSH). Denominator excludes patients who did not consent to linkage of their questionnaire data to an extract of their clinical data, leaving: 766 GU medicine patients and 86 GP-LESSH patients, thus numbers of GP-LESSH patients too small to permit analyses by gender. Corresponding GUMCAD codes are given in parentheses. GU = genitourinary; GP-LESSH = general practice-based Locally Enhanced Services for Sexual Health; STI = sexually transmitted infection; NSU = non-specific urethritis; CT = *Chlamydia trachomatis*; GC = gonorrhoea

In both settings, men were more likely to have acute STI diagnosis/es than women (31.3% versus 20.0%, overall). In GU medicine, the most common diagnosis was non-specific urethritis among men (13.4%), and anogenital warts (first episode) among women (8.1% versus 10.6% among men), the latter also the commonest diagnosis in both male and female GP-LESSH patients (18.8% and 13.0%, respectively). Figure 4 shows the percent of patients diagnosed with STIs during their episode of care by setting.

In both settings, symptomatic patients were more likely to have acute STI diagnosis/es than patients without or unsure as to whether they had symptoms: 35.9% versus 19.5%, respectively, among GU medicine patients; 28.6% versus 20.0%, respectively, among GP-LESSH patients.

#### Management of STI cases in GP-LESSH

A total of 24 diagnoses were recorded among the 19 GP-LESSH respondents diagnosed with acute STIs. Appropriate treatment was prescribed for 21 of these STIs (Supplementary Table S3), while two had first episode anogenital warts and were referred elsewhere for treatment (GU medicine and dermatology). The other was first episode herpes for which no treatment was documented. Two further GP-LESSH patients without STI diagnoses were referred to general practice.

Six GP-LESSH respondents diagnosed with recurrences of anogenital warts or anogenital herpes (data not shown) were recorded as receiving appropriate treatment from the GP-LESSH. Five cases were recorded as ‘epidemiological treatment for suspected chlamydia’ and one case of ‘epidemiological treatment of suspected non-specific genital infection (NSGI)’. All were appropriately treated in GP-LESSH.

Six cases of chlamydia were diagnosed among the GP-LESSH sample, of which three were recorded as having at least one partner tested. Partner(s) of two of these cases were recorded as having been treated for chlamydia.

## DISCUSSION

### Statement of principal findings

The GP-LESSH patients surveyed took longer both to seek *and* to receive care. This may be due in part to the service being open just once a week, and also lower perceived risk by GP-LESSH patients and/or service staff since a smaller proportion of GP-LESSH patients reported symptoms and some sexual risk behaviours. There was no difference by setting in either the proportion of patients reporting previous STI diagnosis/es or having acute STI diagnoses. Almost all GP-LESSH respondents received a genital examination, three-quarters received comprehensive STI testing, and almost all STI cases seen in GP-LESSH were appropriately managed there.

### Relation to wider literature

Our finding of lower risk behaviours among GP-LESSH patients is consistent with national probability survey data comparing women reporting chlamydia diagnoses by place of diagnosis.^19^ Our data also suggest that GU medicine and GP-LESSH may serve people with different needs. For example, men attending GP-LESSH were younger than men attending GU medicine, and less likely to have previously sought sexual health care from a GP-LESSH. This suggests that GP-LESSH may be attractive to such men, challenging previous UK data that suggest that men prefer not to access sexual health care from general practice, although these earlier data refer to different areas of the UK and not specifically to GP-LESSH.^20^ However, at the time of our survey, there was no GU medicine clinic in two of the locations with a GP-LESHH, and local data suggest that geographical access is more important to patients than timely access.^21^

In contrast with previous studies of STI management in unselected general practices,^16,22,23^ we found that patients with suspected STIs received comprehensive investigation in Cornwall's GP-LESSH, and those who had STIs diagnosed in this setting were appropriately managed. It should be noted that the general practices we sampled were not offering STI care within routine practice, rather sexual health sessions in line with an agreed service specification. Other research has shown substantial variation in the delivery of LESSH,^11^ so it cannot be assumed that all GP-LESSH have similar outcomes to those we studied.

### Strengths and weaknesses of the study

Due to convenience sampling our data are not fully representative of the target populations. By focusing on one geographical area of the UK, we also limit the extent to which our results can be generalized. We had planned to compare GU medicine and GP-LESSH in other areas but this was not feasible: in one area because the establishment of a GP-LESSH had been postponed, and for logistical reasons related to the LESSH configuration in another area.^11,12^

Despite reasonable response rates, our sample of GP-LESSH patients was much smaller than that of GU medicine patients. This reflects the much smaller scale of GP-LESSH services than GU medicine clinics (with on average just 7 patients seen per week per practice over the study period, by contrast with an average of 152 patients per week at the main GU medicine clinic in Cornwall^15^) as well as the greater difficulty of recruiting geographically dispersed GP-LESSH services.^24^

While we lacked statistical power to detect some differences, given the current dearth of data on people attending community-based services for sexual health care, we believe that it is important that our data and reproducible methods are disseminated,^17^ so service planners and providers can improve their understanding of the risk profiles, and thus needs, of people attending different types of service in their locality.

### Meaning of the study: possible mechanisms and implications for clinicians and policy-makers

While symptomatic patients in both settings were more likely to have acute STIs diagnosed, a substantial minority of asymptomatic patients were also diagnosed with STIs. Risk assessment and triage strategies that differentiate patients according to the presence/absence of reported symptoms are therefore inappropriate and ill-advised, as others have suggested.^25^ Similarly, while GP-LESSH patients may be less likely to present with symptoms, it should not be assumed that they only require minimal testing, as overall, GP-LESSH patients were just as likely to have acute STIs as GU medicine patients.

While the proportion of men who identified as MSM in GP-LESSH was smaller than in the GU medicine sample, 5.0% is still substantially higher than in the general population.^26^. Furthermore, only four of the six MSM who attended the GP-LESSH studied were recorded as having tested for all of chlamydia, gonorrhoea, syphilis and HIV. We do not have data on whether throat or rectal sampling was done. Our data support the need to offer a full range of services for the highest risk in all settings, or prompt referral where that is not possible, as high-risk individuals do access LESSH. For example, provision of a full range of essential services for MSM, including HIV, syphilis and lymphogranuloma venereum testing and hepatitis B vaccination, needs to be available either on site or through proactive referral.

Variations in demography and geography mean that different localities require different combinations of specialist GU medicine and community-based STI services, which may include different models of primary-care delivered sexual health services.^27^ Our data suggest that the model of GP-LESSH we studied may be helping to meet the needs of patients in Cornwall and assisting in the delivery of the National Strategy,^1^ providing a service beyond that of basic STI care. However, it is also important to recognize that where STI services are not open daily, protocols need to be in place to ensure that people who try to use them are, as a minimum, signposted to service(s) where they can receive appropriate care; informed of the need to seek urgent care; and advised to abstain from sex while they do so. These recommendations apply regardless of setting. Any measure that facilitates prompt access to curative treatment, for example, service-led initiatives that enable patients to receive fast referral to this care, can only increase public health benefit. This illustrates the importance of collaboration in the protocols and operation of local STI services.

### Unanswered questions and future research

As community services expand, it is crucial that they offer comprehensive STI care to their patients. STI service planners must ensure that they understand and monitor their patient population, and more generally, their local population to ensure that their transmission prevention needs are met by local STI services. Failing to meet these needs has the inevitable public health consequence of increased, avoidable STI transmission.^28^

## Appendix

**Supplementary Table S1:** A comparison of patients’ demographics, sexual behaviours, and health-related characteristics by setting (GU medicine versus GP-LESSH) and gender

Gender	Males and females			Males			Females				
Setting	GU medicine	GP-LESSH	*P* value*	GU medicine	GP-LESSH	*P* value*	GU medicine	GP-LESSH	*P* value*	*P* value for gender difference within	
*Denominator*	*933*	*111*		*397*	*43*		*536*	*68*			
Characteristic										GU medicine	GP-LESSH
**Demographic**											
Age			0.494			0.218			0.642	0.001	0.552
<20	20.5%	20.7%		10.9%	20.9%		27.6%	20.6%			
20–24	30.6%	36.0%		30.0%	34.9%		31.1%	36.8%			
25–29	16.5%	19.8%		18.3%	18.6%		15.2%	20.6%			
30–34	8.7%	8.1%		12.9%	14.0%		5.6%	4.4%			
35–39	8.0%	3.6%		9.1%	2.3%		7.1%	4.4%			
40–44	5.1%	5.4%		4.6%	4.7%		5.4%	5.9%			
45+	10.6%	6.3%		14.2%	4.7%		7.9%	7.4%			
Median (lower, upper quartiles)	24 (20, 34)	23 (23, 29)		27 (22, 36)	24 (20, 30)		23 (19, 30)	23 (20, 29)			
White ethnicity	99.6%	96.4%	0.045	99.8%	95.4%	0.037	99.4%	97.1%	0.057	0.161	0.697
**Sexual behavioural**											
Gender of partner(s), past year			0.460			0.685			0.306	<0.001	0.451
Only opposite-sex partner(s)	94.1%	94.1%		89.8%	95.0%		97.1%	93.6%			
Only same-sex partner(s)	4.0%	2.9%		8.5%	5.0%		0.8%	1.6%			
Both opposite- and same-sex partners	1.9%	2.9%		1.7%	0.0%		2.0%	4.8%			
Number of partners, past year			0.153			0.641			0.752	0.023	0.269
1	36.1%	40.2%		31.0%	35.7%		39.7%	43.1%			
2	21.7%	19.6%		17.9%	16.7%		24.3%	21.5%			
3–4	20.4%	23.4%		20.1%	26.2%		20.7%	21.5%			
5–9	16.5%	15.9%		20.9%	21.4%		13.4%	12.3%			
10+	5.4%	0.9%		10.2%	0.0%		1.9%	1.5%			
Median (lower, upper quartiles)	2 (1, 4)	2 (1, 3)		3 (1, 5)	2 (1, 4)		2 (1, 3)	2 (1, 3)			
Number of *new* partners, past year			0.132			0.265			0.133	0.147	0.685
0	19.2%	26.7%		18.2%	23.8%		19.9%	28.6%			
1	30.0%	27.6%		26.0%	31.0%		32.8%	25.4%			
2+	50.8%	45.7%		55.8%	45.2%		47.3%	46.0%			
Median (lower, upper quartiles)	2 (1, 3)	1 (0, 3)		2 (1, 4)	1 (1, 3)		1 (1, 2)	1 (0, 3)			
Number of partners, past 3 months			0.259			0.007			0.352	0.024	0.381
0	9.4%	12.8%		9.5%	9.5%		9.3%	14.9%			
1	61.3%	64.2%		54.1%	71.4%		66.5%	59.7%			
2+	29.3%	22.9%		36.4%	19.1%		24.2%	25.4%			
Median (lower, upper quartiles)	1 (1, 2)	1 (1, 1)		1 (1, 2)	1 (1, 1)		1 (1, 1)	1 (1, 2)			
**Health-related**											
Attended service before			0.162			0.155			0.458	0.108	0.345
No^†^	43.4%	55.5%		46.0%	67.4%		41.6%	47.8%			
Yes for a different episode of care	44.4%	32.7%		40.8%	20.9%		47.1%	40.3%			
Yes for the same episode of care	12.1%	11.8%		13.2%	11.6%		11.4%	11.9%			
Registered with a GP			0.223			0.018			0.437	0.004	0.095
No	3.2%	5.5%		5.6%	9.5%		1.6%	2.9%			
Yes	92.9%	87.3%		89.0%	73.8%		95.7%	95.6%			
I'm not sure	3.8%	7.3%		5.4%	16.7%		2.8%	1.5%			
Ever tested for chlamydia			0.350						0.690	0.003	0.070
No	20.3%	21.1%		28.2%	35.7%	0.288	14.7%	11.9%			
Yes	75.1%	77.1%		65.6%	59.5%		81.8%	88.1%			
Not sure	4.6%	1.8%		6.2%	4.8%		3.5%	0.0%			
Previous STI diagnosis/es			0.663			0.734			0.911	0.197	0.549
No	59.8%	63.9%		61.2%	71.4%		58.7%	59.1%			
Yes	35.5%	34.3%		33.0%	28.6%		37.3%	37.9%			
Not sure	4.7%	1.9%		5.9%	0.0%		3.9%	3.0%			

GU = genitourinary; GP-LESSH = general practice-based Locally Enhanced Services for Sexual Health; STI = sexually transmitted infection

**P* value for difference between settings

^†^‘No’ corresponds to those patients who had never attended the GP surgery before and those patients who had attended the GP surgery but not for a sexual health reason

**Supplementary Table S2:** A comparison of reasons reported for seeking care by setting (GU medicine versus GP-LESSH) and gender

Gender	Males and females			Males			Females		
Setting	GU medicine	GP-LESSH	*P* value*	GU medicine	GP-LESSH	*P* value*	GU medicine	GP-LESSH	*P* value*
*Denominator*	*933*	*111*		*397*	*43*		*536*	*68*	
Reasons for seeking care^†^									
I have (or had) symptoms	38.9%	34.0%	0.406	36.2%	29.3%	0.138	40.9%	37.1%	0.624
My partner has (or had) symptoms	7.9%	4.9%	0.105	11.2%	4.9%	0.274	5.4%	4.8%	0.776
I did not have symptoms but wanted a check-up^‡^	36.3%	47.6%	0.355	36.2%	48.8%	0.244	36.4%	46.8%	0.434
My partner has been diagnosed with an infection and needed to come to the clinic/surgery	4.9%	4.9%	0.995	5.5%	4.9%	0.777	4.4%	4.8%	0.788
Someone from the clinic/surgery called me in	1.2%	0.0%	0.690	1.4%	0.0%	0.699	1.0%	0.0%	0.712
I wanted a HIV test	7.1%	4.9%	0.411	8.7%	0.0%	0.621	5.8%	8.1%	0.476
My GP or practice nurse told me to come here	13.0%	10.7%	0.774	10.6%	7.3%	0.495	14.7%	12.9%	0.860
My symptoms have not gone away since I last came here for treatment	5.2%	5.8%	0.678	6.3%	4.9%	0.744	4.4%	6.5%	0.084
Last time I came here someone asked me to come back for more treatment or for another check-up /test	6.8%	4.9%	0.753	4.6%	7.3%	0.696	8.5%	3.2%	0.353
Other reason(s)	4.9%	1.9%	0.020	3.3%	0.0%	0.698	6.0%	3.2%	0.099

GU = genitourinary; GP-LESSH = general practice-based Locally Enhanced Services for Sexual Health

**P* value for difference between settings

^†^Patients could report more than one reason for seeking care. Response options were presented in the same order in both questionnaires

^‡^This does not correspond to the proportion of asymptomatic screens carried out because asymptomatic patients sometimes reported other reasons for attendance, e.g. wanting an HIV test

**Supplementary Table S3:** Treatment prescribed/care recorded for GP-LESSH patients diagnosed with acute STIs*

Study number	STI or epidemiological treatment recorded	Treatment/care recorded	Management regarded as appropriate?
3010	Anogenital warts, first attack Epi treatment of suspected NSGI	Azithromycin stat 1 g Warticon 4 weeks – review	Appropriate treatment
3021	Chlamydial infection Anogenital warts first attack Epi treatment of suspected CT	Azithromycin stat 1 g Warticon 4	Appropriate treatment
3022	Chlamydial infection	Azithromycin stat 1 g	Appropriate treatment
3034	Anogenital warts, first attack	Warticon	Appropriate treatment
3038	Non-GU/NSU/treatment of mucopurulent cervicitis in females Anogenital warts, first attack	Referred to GU medicine	Referral for care
3049	Anogenital warts, first attack	None recorded	No treatment recorded
3102	Anogenital HSV, first attack	Aciclovir 5 days	Appropriate treatment
3105	Chlamydial infection	Doxycycline 100 mg twice daily 2 weeks Metronidazole 400 mg twice daily 1 week	Appropriate treatment
3112	Chlamydial infection	Zithromax 1 g 1 day	Appropriate treatment
3115	Anogenital warts, first attack	Cryotherapy	Appropriate treatment
3117	Chlamydial infection PID	Doxycycline 2 weeks Metronidazole 1 week	Appropriate treatment
3203	Anogenital warts, first attack	Cryotherapy	Appropriate treatment
3215	Anogenital warts, first attack	Cryotherapy	Appropriate treatment
3219	Chlamydial infection	Azithromycin	Appropriate treatment
3221	Anogenital HSV, first attack Epi treatment of suspected CT	Azithromycin	Unclear for HSV; appropriate treatment for CT
3224	Anogenital warts, first attack Epi treatment of suspected CT	Azithromycin Cryotherapy	Appropriate treatment
3229	Anogenital warts, first attack	Warticon Metronidazole gel	Appropriate treatment Unclear – possible treatment for BV
3230	Anogenital warts, first attack	Warticon Referral to dermatology	Appropriate treatment and referral for care
3231	Anogenital warts, first attack	Warticon	Appropriate treatment

GU = genitourinary; GP-LESSH =general practice-based Locally Enhanced Services for Sexual Health; STI = sexually transmitted infection; NSU = non-specific urethritis; HSV = herpes simplex virus; BV = bacterial vaginosis; CT = chlamydia; NSGI = non-specific genital infection

*Only patients with acute STI diagnosis/es are included in this table. One patient was excluded as they had ‘Advice re. molluscum’ recorded in their notes. Another was recorded as ‘for psoriasis – balanitis’ in their notes. A further five patients were recorded as receiving ‘epidemiological treatment of suspected CT’ – all of whom received appropriate medication. A further six patients are excluded from this table as they were recorded as having ‘anogenital HSV: recurrence’ (*n* = 4) or ‘anogenital warts: recurrence’ (*n* = 2)

## References

[1] Department of Health. The National Strategy for Sexual Health and HIV. London, UK: SAGE Publications, 2001

[2] Medical Foundation for AIDS and Sexual Health. Standards for the Management of Sexually Transmitted Infections (STIs). London: SAGE Publications, 2010

[3] Medical Foundation for AIDS and Sexual Health. Recommended Standards for Sexual Health Services. London: SAGE Publications, 2005

[4] NHS Information Standards Board. Data Standards: 48 Hour Genitourinary Medicine Access Monthly Monitoring (GUMAMM). London: SAGE Publications, 2007

[5] Simms I, Talebi A, Rhia J, The English National Chlamydia Screening Programme: variations in positivity in 2007/2008. Sex Transm Dis 2009;36:522–7 19455079 10.1097/OLQ.0b013e3181a2aab9

[6] Department of Health, RCGP Sex Drugs and HIV Task Group, BASHH, Faculty of Family Planning and Reproductive Health, RCN, and NANCSH. Competencies for Providing More Specialised Sexually Transmitted Infection Services within Primary Care. London: SAGE Publications, 2005

[7] Department of Health, BASHH, Faculty of Family Planning and Reproductive Health, NANCSH., RCGP Sex Drugs and HIV Task Group, and RCN. Competencies for Providing More Specialised Sexually Transmitted Infection Services within Primary Care: Assessment Toolkit. London: SAGE Publications, 2008

[8] RCGP Sex Drugs and HIV Task Group and BASHH. Sexually Transmitted Infections in Primary Care. London: SAGE Publications, 2006 Report No.: 1st edition

[9] RCGP Sex Drugs and HIV Task Group. Minimum Standards of Care for Sexual Health in Primary Care. London: SAGE Publications, 2002

[10] The NHS Confederation and BMA. Investing in General Health. The New General Medical Services Contract. London: SAGE Publications, 2003

[11] Bailey AC, Johnson SA, Cassell JA Are primary care-based sexually transmitted infection services in the UK delivering public health benefit? Int J STD AIDS 2010;21:39–45 20029064 10.1258/ijsa.2009.008461

[12] Yung M, Denholm R, Peake J, Distribution and characteristics of sexual health services provision in primary and community care in England. Int J STD AIDS 2010;21:650–2 21097740 10.1258/ijsa.2010.010198

[13] Payne N, O'Brien R Health Economics of Sexual Health: A Guide for Commissioning and Planning. London: SAGE Publications, 2005

[14] Health Protection Agency. *Genitourinary Medicine Clinic Activity Dataset (GUMCAD).* See www.hpa.org.uk/web/HPAweb&Page&HPAwebAutoListName/Page/1201094610372 (last checked 18 January 2012)

[15] Department of Health. *48 hour Genitourinary Medicine Access Monthly Monitoring (GUMAMM) Statistics* See www.dh.gov.uk/en/Publicationsandstatistics/Statistics/Performancedataandstatistics/SexualHealth/index.htm (last checked 18 January 2012)

[16] Neale R, Keane F, Saulsbury N, Who attends primary care services prior to attendance at genitourinary services and what level of care have they received? Sex Transm Inf 2008;84:233–4 10.1136/sti.2007.028498 18283092

[17] Aicken CR, Cassell JA, Estcourt CS, Rationale and development of a survey tool for describing and auditing the composition of, and flows between, specialist and community clinical services for sexually transmitted infections. BMC Health Serv Res 2011;11:30 21306604 10.1186/1472-6963-11-30 PMC3045289

[18] Stata Statistical Software: Release 10 [Computer Program]. College Station, TX, USA: SAGE Publications, 2007

[19] Cassell JA, Mercer CH, Fenton KA, A comparison of the population diagnosed with chlamydia in primary care with that diagnosed in sexual health clinics, and its implications for a national screening programme. Public Health 2006;120:984–988 16949114 10.1016/j.puhe.2006.05.025

[20] French RS, Gerressu M, Griffiths C, and the One Stop Shop Evaluation Team. Evaluation of One-Stop Shop Models of Sexual Health Provision. London: SAGE Publications, 2008

[21] Kehinde R, Keane FE, McNicol L Access to STI services: What Matters Most – Speed or Distance? Int J STD AIDS 2011;22:133

[22] Nicholson A, Rait G, Murray-Thomas T, Management of first-episode pelvic inflammatory disease in primary care: results from a large UK primary care database. BJGP 2010;60:756–62 10.3399/bjgp10X532404 PMC2944949 20883614

[23] Nicholson A, Rait G, Murray-Thomas T, Management of epididymo-orchitis in primary care: results from a large UK primary care database. BJGP 2010;60:763–9 10.3399/bjgp10X532413 PMC2944950 20883615

[24] Slater W, Sadler K, Cassell JA, Horner P, Low N What can be gained from comprehensive disaggregate surveillance? The Avon surveillance system for sexually transmitted infections. Sex Transm Inf 2007;835:411–5 10.1136/sti.2006.023440 PMC2659036 17344247

[25] David N Are they really asymptomatic? Int J STD AIDS 2008;19:575–6 18663054 10.1258/ijsa.2008.008159

[26] Johnson AM, Mercer CH, Erens B, Sexual behaviour in Britain: partnerships, practices, and HIV risk behaviours. Lancet 2001;358:1835–42 11741621 10.1016/S0140-6736(01)06883-0

[27] Dabrera G, Johnson S, Bailey A, Kinross P, Cassell J Mapping of Sexual Health Services for Men who have Sex with Men (MSM). See: http://www.hpa.org.uk/webc/HPAwebFile/HPAweb_C/1317137242739 (last checked 18 December 2012)

[28] White PJ, Ward H, Cassell JA, Mercer CH, Garnett GP Vicious and virtuous circles in the dynamics of infectious disease and the provision of health care: gonorrhea in Britain as an example. J Infect Dis 2005;192:824–36 16088832 10.1086/432004

